# Bringing nutritional ketosis to the table as an option for healing the pediatric brain

**DOI:** 10.3389/fnut.2024.1408327

**Published:** 2024-06-12

**Authors:** Tracy S. Gertler, Robyn Blackford

**Affiliations:** ^1^Division of Pediatric Neurology, Ann & Robert H. Lurie Children’s Hospital of Chicago, Chicago, IL, United States; ^2^Division of Clinical Nutrition, Ann & Robert H. Lurie Children’s Hospital of Chicago, Chicago, IL, United States

**Keywords:** ketogenic diet, pediatrics, child neurology, ADHD, autism spectrum disorder, mental health

## Abstract

Our core premise is that personalized variations of a ketogenic diet are likely to benefit pediatric patients with neuropsychiatric symptoms across multiple domains. Although pediatric epilepsy is currently a well-accepted indication for a strict ketogenic diet, there is a dearth of knowledge and therefore clinical guidelines upon which to recommend nutritional ketosis for pervasive pediatric conditions such as autism spectrum disorder and ADHD, even when comorbid epilepsy is present. However, there are published cohort studies and current clinical trials implementing medical ketogenic therapies for cognitive impairment, psychiatric comorbidities, motor disability, and even neuroinflammation. As holistic practitioners, it is imperative that we consider the health of a child in its entirety - and additionally offer the ketogenic diet as a therapeutic option when it may be synergistic in treating extra-neurologic diseases such as obesity. While there are uniquely pediatric potential adverse side effects such as linear growth deceleration and micronutrient deficiencies, previous trials in epilepsy and our center’s experience have already proven the ketogenic diet to be a low-risk intervention when optimized with appropriate patient monitoring and support.

## Introduction

Multiple epidemiologic studies agree that the burden of pediatric mental health diagnoses is exponentially increasing (1). Commonly cited explanations are diverse, ranging from increased use of and dependence on social media, increased obesity rates, and decreased medical insurance coverage for mental health support. Yet, while additional funding and availability of psychopharmacological therapies and mental health practitioners clearly help, current evidence-based approaches are not enough. Classical medical therapies often have severe and lifelong adverse side effects, especially for the developing brain. Improvement to the desired level of independent functioning frequently demands polypharmacy, further amplifying the risk of side effects.

Use of a ketogenic diet as a medical therapy for pediatric epilepsy was first described by Dr. Russell Wilder in the Mayo Clinic Proceedings (2). Since that time, support for its efficacy at or above the level of standard antiseizure medications has solidified its keystone role (3). In conjunction with Dr. Wilder, a pediatrician Mynie Peterman noted parallel improvements in behavior and cognition on a ketogenic diet (4). However, these benefits are often seen as secondary, beneficial only as compared to the lack of an adverse outcome, or simply fortuitous. *Our perspective is that this benefit needs to be brought front and center as a primary outcome, so that we can identify children with neurodevelopmental impairments, with or without seizures, who may require it most.*

**Figure 1:**
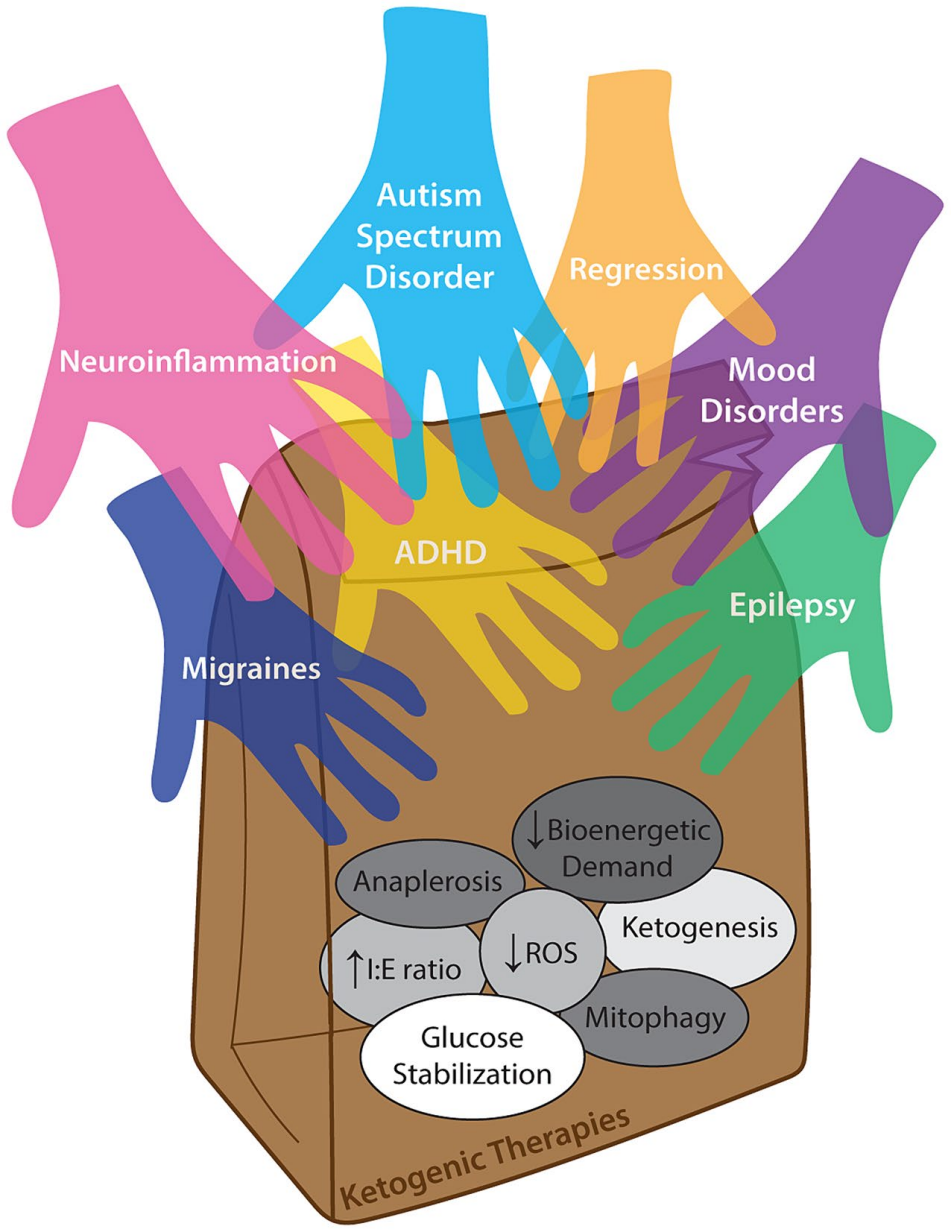
Concept illustration of multiple neuropsychiatric diagnoses which may benefit from (i.e., reaching into a lunch bag representing) ketogenic therapies. The diversity of proposed mechanisms of action that may confer different benefits across a range of clinical contexts are inside the bag, and while it is likely that different diagnoses improve secondary to varied combinations of these mechanisms, direct causation for any single disease is not definitively known. ‘Anaplerosis’ is refilling of TCA cycle intermediates. “Decreased bioenergetic demand” is improving mitochondrial function. “Increased I:E ratio” is a relative increase in inhibitory (e.g., GABAergic) signaling over excitatory (glutamatergic) transmission. “Decreased ROS” is reduction of radical oxygen species. “Ketogenesis” is the actual generation of ketone bodies. “Glucose stabilization” is decreasing glucose variability over time. “Mitophagy” is recycling and generation of healthier mitochondria (as reviewed by Masino and Rho (8)).

Within the subtext of epilepsy studies, there is evidence ranging from anecdotal to statistically-significant secondary outcomes that improvements in other domains besides epilepsy are present. This is highly relevant, as ~80% of children with epilepsy have comorbid behavioral or cognitive impairment (5) and the highest risk factor for autism spectrum disorder in children with epilepsy is intellectual disability (6). Given the cascade of paradigm-shifting studies in adult psychiatric disease, it stands to reason that children may also benefit from a ketogenic diet or nutritional ketosis (7) – and given the existing expertise in pediatric epilepsy centers, pediatric practitioners are well-equipped to initiate and study this powerful intervention. We acknowledge that evidence-based use of the ketogenic diet in pediatrics is currently limited to intractable epilepsy; however, we suggest that “off-label” indications are supported by anecdotal evidence while the academic community mobilizes to initiate objective, randomized controlled trials with standardized, quantitative outcome measures to improve the standard of care for multiple common neuropsychiatric conditions. Although review of proposed mechanisms of the ketogenic diet is beyond the scope of this paper, we suggest that the multifactorial, pleiotropic effects of the ketogenic diet in different clinical contexts converge upon and pave the way for a common therapeutic improvement for an increasingly broadening range of common neuropsychiatric conditions (Figure 1).

## Application of a ketogenic diet as a medical therapy for pediatric mental health

While a strict 4:1 classic ketogenic diet may be necessary for drug-resistant epilepsy (9), recent studies suggest a less-restrictive Modified Atkins Diet is often sufficient to yield demonstrable benefit (10, 11). If the goal of using a ketogenic diet is to improve a child’s mental health so that they more easily socialize and learn in parallel with other children in a regular school environment, a comparatively less restrictive protocol that can be initiated at home rather than during an inpatient hospital admission is ideal. The effective and therapeutic degree of ketosis in an individual is exquisitely patient-specific and does not correlate perfectly to the ketogenic diet ratio across individuals. An intervention with a higher degree of flexibility and reduced risk for side effects is more likely to be successful in the long-term.

There are several versions of a ketogenic diet available for use. Our version of the Modified Atkins Diet (MAD), also called Modified Ketogenic Diet (MKD), consists of 40–60 g total carbohydrate per day with 1–2 tablespoons fat added to each meal. Fat sources are often butter, mayo, and oil. If feasible for patients, MCT oil is the preferred fat source and is encouraged at each meal. MCT oil will bypass the usual fat metabolism to be used more immediately for conversion to ketones, thus expediting ketogenesis by bypassing digestive enzymes and the carnitine shuttle (12). In addition, use of MCT oil may allow for a comparatively higher carbohydrate and protein intake without compromising nutritional ketosis (3).

## Uniquely pediatric indications, contraindications, and side effects

A challenge that’s imperative upon all pediatric practitioners is to identify inborn errors of metabolism that benefit from specific dietary therapies as early as possible. A subset of these (e.g., phenylketonuria) are readily identified on the Newborn Screen, a primarily biochemical screening test with modest state-to-state variability. However, other syndromes are not able to be screened for in a blood spot or do not emerge until later in infancy/childhood. For example, Glut1 deficiency syndrome is a genetic-metabolic condition that impairs transport of glucose across the blood brain barrier; it is diagnosed by identification of pathogenic variants in *SLC2A1* and/or low absolute or relative glucose in the cerebrospinal fluid, in the proper clinical context. Most children do not develop symptoms until early infancy or later, and have broad phenotypic variability ranging from severe epilepsy to disruptive movement disorders and mild–moderate intellectual disability (13). Because a ketogenic diet improves not only seizure control but also cognitive development, motor function, and freedom from disabling hemiplegic migraines, it is critical that testing is made available early in life and with minimal invasiveness when possible (i.e., genetic or blood screening rather than diagnostic lumbar puncture). Similarly, other genetic/metabolic diagnoses where a ketogenic diet is considered a potential first-line therapy because glucose supply to the brain is impaired include glycogen storage disorders (type III/Forbes’ disease and type V/McArdle’s disease), phosphofructokinase deficiency, pyruvate dehydrogenase complex deficiency, and some mitochondrial disorders (14). In contrast, there are specific inborn errors of metabolism where implementation of a ketogenic diet is contraindicated and would be dangerous, including: carnitine deficiency (primary), carnitine palmitoyltransferase (CPT) I or II deficiency, carnitine translocase deficiency, beta-oxidation deficiencies including medium-chain acyl dehydrogenase deficiency (MCAD), long-chain acyl dehydrogenase deficiency (LCAD), short-chain acyl dehydrogenase deficiency (SCAD), long-chain 3-hydroxyacl-CoA deficiency, medium-chain 3-hydroxyacl-CoA deficiency, pyruvate carboxylase deficiency and porphyria (15). We bring these up to suggest that a recommendation to start a ketogenic diet as well as metabolic and genetic testing should be thoughtfully considered (see Table 1 for suggested testing) by a knowledgeable healthcare team, regardless of the indication (though this often is linked to the etiology).

**Table 1:** Recommendations for testing.

Demographics	Serum studies	Functional studies (indication)	Tests to consider to exclude other diagnoses (symptom)
Height Weight BMI Head circumference	CBC BMP Magnesium Ionized calcium Lactic acid Lipid Profile Fasting Insulin BHB Carnitine level Acylcarnitine profile Vit D levels Urine fractional calcium excretion	EEG (seizures) MR brain/spine (demyelinating, IEM) Vanderbilt survey (ADHD) PHQ9, BDI (MDD) GAD7 (GAD) Disease-specific parental questionnaires CORE-KETO outcomes	Thiamine (B1) level (memory deficit) Vit B12 level (psychosis) Copper, ceruloplasmin (psychosis) Lead level (psychosis) Thyroid function tests (mood changes, psychosis, regression) Chromosomal microarray and/or Whole Exome Sequencing (developmental delay, autism spectrum disorder) CSF glucose and lactate (developmental delay +/− seizures and movement disorder) CSF folate, neopterin, tetrahydrobiopterin (developmental delay)

Recommendations for testing before and concurrent with ketogenic diet initiation. Demographic information and basic measurements are indicated for following growth. Serum studies are needed to monitor electrolyte, vitamin, and hormonal changes that may indicate risk of side effects (e.g., nephrocalcinosis). Functional studies are expected to be disease-specific, but suggestions provided including clinical monitoring relevant to epilepsy, demyelinating disorders, ADHD, mood disorders, and consensus diet guidelines. Finally, dependent upon the clinical symptoms being addressed, we suggest careful survey of other causes of neuropsychiatric symptoms that are commonly missed at first assessment and would require different treatment protocols.

Parents often wish to know potential adverse effects of a ketogenic diet in parallel with feasibility considerations. During the transition period onto a ketogenic diet, common issues such as dehydration, relative hypoglycemia, nausea, and decreased energy can be addressed with increased consumption of fluid-containing electrolytes, and quickly resolve. Further corrections for abnormal electrolytes, metabolic acidosis, vitamin deficiencies, and constipation are possible. Historically, negative pediatric health outcomes have included linear growth deceleration, increased gastroesophageal reflux and constipation, and increased risk of nephrocalcinosis, with some consideration for increased risk of osteoporosis and secondary carnitine deficiency (with decreased efficacy of a ketogenic diet and/or associated cardiomegaly), and hepatotoxicity in adulthood. In our experience, these side effects are rare when the ketogenic diet is initiated as a medical therapy and followed by an experienced dietitian in even the youngest patients (16). Using a more liberal ketogenic diet, such as the MAD/MKD that brings about a lower level of nutritional ketosis, will lower the risk of potential side effects (11). As many children take liquid medication with a high carbohydrate content, dose formulation must be considered and often adjusted to crushed tablets or opened capsules; parents must also be aware of antibiotics, antipyretics, and common supplements that may be suggested by physicians and available over the counter in sugary syrups. Overall, the highest risk is perhaps parental dissatisfaction or inability to adhere to a ketogenic diet, prompting discontinuation. For example, implementation of a ketogenic diet in children with intractable epilepsy has been demonstrated to increase parental stress (17). In an ideal situation, a multidisciplinary team is able to provide parental education and support to initiate and maintain the diet with the understanding that it is a major lifestyle change impacting the entire family; an unfortunate reality is that this may not be possible for many biopsychosocial reasons in all cases where it is medically indicated.

## Existing literature in pediatric mental health

Given the number of children who have been treated with a ketogenic diet for epilepsy, what can we learn about *other* changes that they have experienced in their health in parallel? In two studies from Johns Hopkins, parental goals for starting a ketogenic diet cited improvement in cognition as the second most common reason for initiating the consult, second only to potential reduction in seizure frequency (18, 19). Remarkably, this goal was achieved in more than 50% of families. In a cohort of fifty children from the Netherlands, a positive cognitive and behavioral impact was noted in children with refractory epilepsy treated with a ketogenic diet as compared to a control group over a 4 month period, independent of any changes in seizure frequency (20). Parental questionnaires reflected lowered anxiety, mood-disturbed behavior, and higher productivity; in parallel, objective cognitive tests demonstrated improved receptive vocabulary and reaction time in the absence of any medication changes. If and when a ketogenic diet may be recommended for adolescent mood disorders is an active area of research with initial promise (21, 22). A key question moving forward is what are the optimal measures by which to quantify improved mood and cognition, as well as other non-seizure outcomes.

### ADHD

Approximately 10% of children in the US (6 million) are currently diagnosed with Attention Deficit Hyperactivity Disorder (ADHD), making it the most common neuropsychiatric condition of our time (23). ADHD is also the most common comorbid psychiatric diagnosis in children with epilepsy, affecting nearly 30% of patients (24). This overlap begs the question of whether intractable ADHD may benefit from similar therapies known to be effective in intractable epilepsy. Intriguingly, children with ADHD but not epilepsy are also noted to have more frequent interictal epileptiform discharges on sleep-deprived electroencephalograms (EEGs) (25). In children who are able to maintain a ketogenic diet for intractable epilepsy for a year, social problems and attention have been improved in parallel with seizure reduction (26).

It is worth noting that higher overall sugar consumption, especially in liquid form, is commonly thought to increase symptoms of hyperactivity and inattention and has support in primary and meta-analytic studies (27, 28), though isolating other socioeconomic and lifestyle factors that contribute to dietary choices is difficult (29). Multiple ‘restrictive’ diets including the DASH diet, Mediterranean diet, and a strict elimination diet offered some benefit in limited cohort samples, though sustainability remains a concern, and common elements such as reduction of processed food intake are pervasive confounding factors.

The association between multiple micronutrient deficiencies and severity of ADHD symptoms is well-documented; low magnesium, iron, and zinc levels all correlate with increased diagnosis rates and symptom severity scales, while supplementation with these as well as vitamin B6 (pyridoxine), and omega-3 fatty acids have shown benefit (30–33). Use of probiotics and avoidance of allergens triggering autoinflammatory responses continue to accumulate and are worth mentioning, though beyond the scope of this perspective. However, we propose that there is a role for tailoring a ketogenic diet to children who live with ADHD despite correction of underlying medical issues and nutritional deficiencies, school accommodations and psychosocial education, and either first-line medical management or contraindications and side effects to stimulant use.

### ASD

Approximately 20% of children with epilepsy are also diagnosed with autism spectrum disorder (ASD) (34), suggesting shared neurobiological underpinnings. Co-occurring diagnoses also introduce challenges in using certain antiseizure medications where adverse side effects such as behavioral shifts, mood changes, or hyperactivity are amplified. Excluding data from animal models, there is limited evidence thus far that nutritional ketosis can be beneficial. For example, a 6 month pilot study of a ketogenic diet using primarily MCT oil in 30 children showed improvement in a majority of them on the Childhood Autism Rating Scale (CARS), though was limited by a 40% non-compliance rate (35). Multiple anecdotal reports suggest potential improvement across cognitive, behavioral, and psychiatric domains (36, 37). However, larger-scale standardized studies are sorely needed. It is clear that antipsychotic medications commonly used to address aggression in children with autism present an increased risk of metabolic syndrome, whereas nutritional ketosis maintains or improves the cardiovascular and metabolic health of most individuals. Conceptually, if impaired neuronal metabolism contributes to symptoms, alleviating rather than exacerbating underlying metabolic stress would appear beneficial, though the data are not yet available to demonstrate this.

### Regression

Exciting data are emerging to suggest a role for the ketogenic diet in Alzheimer’s Disease (AD), referred to by some as “Type III Diabetes Mellitus” given demonstration of glucose hypometabolism on PET imaging (38). We suggest that children with Down Syndrome/Trisomy 21 may warrant special consideration for a ketogenic diet, given increased expression of alpha-synuclein and a well-established genetic predisposition for early-onset dementia similar etiologically to AD. Case reports thus far are encouraging (39). Similarly, Down syndrome regression disorder (DSRD) is a syndrome in which neurological functioning across domains becomes impaired in a multifactorial yet poorly understood cascade of autoimmune, proconvulsant, and perhaps neurodegenerative reactions (40). While IVIG offers some suggested benefit, we propose a role for nutritional ketosis as a uniquely beneficial therapy because it is pleotropic (and thus likely to address multiple disease mechanisms), and is a low-risk intervention compared to long-term steroids, antipsychotic medication, or immunosuppression.

## Case studies

As we expand our practice to include a broader swathe of neuropsychiatric diagnoses, we continue to draw from our past experiences with children with intractable epilepsy. Below are case examples where other appreciable benefits of a ketogenic diet were clearly noted.

### Patient A

Patient A is a 21 year old young man with Dravet Syndrome. He presented at 5 months of age with seizure onset and subsequent diagnoses of intractable epilepsy, intellectual disability, behavioral concerns, and gait abnormalities. Initial seizures included hemi- and generalized convulsions that frequently progressed to status epilepticus. At 9 months, he began having myoclonic jerks and absence seizures. He was trialed on 6 antiseizure medications, yet continued to have persistent daily seizures. He was started on the classic ketogenic diet as an outpatient at 19 years old, and fed by gastrostomy tube 95% of the time with small tastes by mouth. His ratio was increased slowly over a few months to a final ketogenic ratio of 2.5:1. At that time, his mother noted a slight decrease in the number of seizures, reduced use of rescue medication, and less intense convulsions; however, they were still occurring daily. Due to difficulties in obtaining the lower ratio formula, they started to use ketogenic formula in the 4:1 ratio. His seizures were reportedly better on the higher ratio and worsened when he went back on the lower ratio formula, so it was decided to maintain the 4:1 ratio. His mother reported that after several months of the higher ratio formula, cognitive improvements unexpectedly emerged; he was able to speak in full sentences and follow multiple step commands, which was not possible prior to the start of the diet therapy. *We present this case as an example where seizure improvement was modest, yet expressive and receptive language gains were remarkable.*

### Patient B

Patient B is an 11 year old boy with Angelman Syndrome, evidenced by characteristic facial features, EEG features, and a pathogenic *UBE3A* gene variant. He was diagnosed with febrile seizures in infancy, then subsequently with unprovoked, atypical absence seizures requiring an antiseizure medication. Subsequent atonic seizures prompted the addition of a second antiseizure medication. He had many improvements at this time, such as taking steps, sleeping, and improved PO intake as well as seizure remission. At 3 years old, seizures increased again, and a third antiseizure medication was required. The classic ketogenic diet was initiated at 4 years old when seizure frequency was more than 10 times per day; seizures stopped within a few days of initiation, and he is maintained on a KD at a 3:1 ratio and fully fed by mouth. While his seizures are very well controlled on KD, his parents report that he is more alert, engaged with his surroundings, and social with family and friends. *Taken together, gains in cognition and behavior have provided improved quality of life in parallel with seizure remission.*

### Patient C

Patient C is a 4 year old boy with severe intellectual disability, spastic quadriparesis, optic nerve hypoplasia, congenital hypothyroidism, and intractable multifocal epilepsy secondary to a pathogenic variant in *IMPDH2.* Seizures began at 2 months of age, prompting initiation of an antiseizure medication with subsequent developmental gains. Abnormal eye movements prompted initiation of a second antiseizure medication. With onset of status epilepticus, a third antiseizure medication was added. At this time, his parents noted an asymmetric gait with dragging of his left leg, and expressive speech regression, as he had become nonverbal and communicated only with an assistive tablet. He became easily aggravated, with hair pulling and frequent hitting his own head and others. Patient C started the classic ketogenic diet at 3 years old. He received food by mouth during the day and took water and one formula feeding by gastrostomy tube during the night. On a 3:1 ketogenic ratio, metabolic acidosis was noted on routine labs and seizures did not improve. However, his mother reported that he is more alert and active after starting the KD. His therapists noted a longer attention span and restoration of his verbal skills to near baseline. *Medications are still adjusted in conjunction with the ketogenic diet to optimize management of epilepsy as well as cognitive and motor performance.*

### Concluding remarks

In this article, we provide a rationale and supporting existing evidence for use of a ketogenic diet in pediatric neuropsychiatric conditions and provide limited case studies from our own experience where its implementation provided clear benefits. In the future, we expect nutritional ketosis to be more commonly offered and trialed given its advantageous risk–benefit profile. While epilepsy remains the primary evidence-based indication for its use in pediatric neurology, small cohort studies and open-label trials have already opened the door to its potential role as supplemental therapies in migraine headaches, demyelinating disease, and brain tumors. We expect that the widespread need for additional solutions for ADHD, ASD, and intellectual disability coupled with our existing experience and best practices in this sphere will usher in an opportunity for earlier, more frequent consideration of nutritional ketosis.

## Data availability statement

The original contributions presented in the study are included in the article/supplementary material, further inquiries can be directed to the corresponding author.

## Author contributions

TG: Writing – original draft, Writing – review & editing. RB: Writing – original draft, Writing – review & editing.
